# New Hepatic Resection Criteria for Intermediate-Stage Hepatocellular Carcinoma Can Improve Long-Term Survival: A Retrospective, Multicenter Collaborative Study

**DOI:** 10.31557/APJCP.2020.21.10.2903

**Published:** 2020-10

**Authors:** Hiroya Iida, Masaki Kaibori, Fumitoshi Hirokawa, Yoshihiro Inoue, Masaki Ueno, Kousuke Matsui, Morihiko Ishizaki, Shogo Tanaka, Shigekazu Takemura, Takeo Nomi, Daisuke Hokutou, Takehiro Noda, Hidetoshi Eguchi, Takuya Nakai, Hiromitsu Maehira, Haruki Mori, Masaji Tani, Shoji Kubo

**Affiliations:** 1 *Department of Surgery, Shiga University of Medical Science, Otsu, Japan. *; 2 *Department of Surgery, Kansai Medical University, Hirakata, Japan. *; 3 *Department of General and Gastroenterological Surgery, Osaka Medical College, Takatsuki, Japan. *; 4 *Second Department of Surgery, Wakayama Medical University, Wakayama, Japan. *; 5 *Department of Hepato-Biliary-Pancreatic Surgery, Osaka City University Graduate School of Medicine, Osaka, Japan. *; 6 *Department of Surgery, Nara Medical University, Kashihara, Japan. *; 7 *Department of Gastroenterological Surgery, Graduate School of Medicine, Osaka University, Suita, Japan. *; 8 *Department of Surgery, Faculty of Medicine, Kinki University, Osaka-sasayama, Japan. *

**Keywords:** Hepatocellular carcinoma, BCLC stage B, intermediate stage, hepatectomy, hepatic resection

## Abstract

**Background:**

Hepatic resection (HR) is not recommended for intermediate-stage hepatocellular carcinoma (HCC) by the Barcelona Clinic Liver Cancer criteria. We examined the prognostic factors of HR for intermediate-stage HCC and developed new HR criteria for intermediate-stage HCC.

**Methods:**

A total of 110 patients who underwent HR without any prior treatment for intermediate-stage HCC between January 2007 and December 2012 were enrolled at eight university hospitals. The outcomes and prognostic factors of HR were evaluated to develop new HR criteria.

**Results:**

In terms of tumor size and number, the most significant prognostic factors were within the up-to-seven criteria. Furthermore, serum albumin level ≥35 g/L and serum alpha-fetoprotein (AFP) level <100 µg/L were prognostic factors in multivariate analysis. The 5-year cumulative survival rate of patients based on the new HR criteria (i.e., up-to-seven, AFP <100 µg/L, albumin ≥35 g/L) was 81.4% and the area under the curve was 0.653.

**Conclusions:**

HR can improve the prognosis of patients with intermediate-stage HCC who meet the up-to-seven criteria and have serum albumin levels ≥35 g/L and serum AFP levels <100 µg/L. These factors could serve as new HR criteria for intermediate-stage HCC.

## Introduction

Approximately 0.7 million people develop hepatocellular carcinoma (HCC) annually worldwide (Forner et al., 2012; Bruix et al., 2014). HCC is a common disease in Asia and Africa and shows an increasing trend in Europe and America (Torre et al., 2015). With the establishment of staging systems and treatment guidelines that stratify patients according to tumor characteristics, such as tumor size and number, and patient conditions, including patient background and hepatic functional reserve, the prognosis of patients with HCC has improved. Moreover, the particular systems and treatment guidelines have provided better knowledge that helps to predict prognosis more accurately and to select the most appropriate treatment approach (de Lope et al., 2012; Kudo et al., 2016). The Barcelona Clinic Liver Cancer (BCLC) staging system is most commonly used for HCC staging and developing therapeutic strategies (Yamakado and Kudo, 2014). This system primarily includes the following stages according to patients’ physical status (PS) and Child-Pugh score: stage 0 (PS 0, Child-Pugh A), stage A–C (PS 0–2, Child-Pugh A–B), and stage D (PS >2, Child-Pugh C). According to the tumor size and number, presence/absence of portal invasion, and PS, stage A (early) is characterized by single or three nodules ≤3 cm, PS 0; stage B (intermediate) by multiple nodules, PS 0; and stage C (advanced) by portal invasion, extrahepatic metastasis, and PS 1–2. Therefore, the range of the intermediate-stage HCC is quite extensive and the need for some subclassification of the BCLC staging system has been advocated (Yamakado and Kudo, 2014; Bolondi et al., 2012; Kudo et al., 2015; Hiraoka et al., 2016).

For intermediate-stage HCC, only transcatheter arterial chemoembolization (TACE) is recommended as the standard treatment option in the BCLC staging system. Hepatic resection (HR) is indicated only for patients with early-stage HCC (BCLC A) with satisfactory liver function. In Japan, although TACE is the first-line treatment for patients with HCC in the intermediate-stage, local treatments, such as HR or radiofrequency ablation (RFA), are also recommended depending on hepatic functional reserve and tumor number and size (The Japan Society of Hepatology, 2013). Recently, several reports showed the effectiveness of HR for intermediate-stage HCC (Torzilli et al., 2013) (Zhong et al., 2013; Zhong et al., 2014; Ho et al., 2016). However, the indication of HR for intermediate-stage HCC is still unclear. In this study, we attempted to develop new resection criteria for intermediate-stage HCC considering tumor stage, biological malignancy of HCC, and liver function. In particular, the novelty of this study is that it was conducted using several liver transplantation criteria.

## Materials and Methods

The subjects were 112 patients with intermediate-stage HCC who underwent curative HR without any prior treatment, such as RFA, TACE, hepatic arterial infusion therapy, or systemic chemotherapy, between January 2007 and December 2012 at eight university hospitals. Two patients who died postoperatively were excluded. Thus, 110 patients were included in the final analysis. Data were obtained retrospectively. This multicenter, retrospective study conformed to the Clinical Research Guidelines and was approved by the ethical committee of each institution (approval number in principal institution: 29-086). Informed consent was obtained by opt-out from all patients or members of their families at each institution. 

The following factors were evaluated: age, sex, presence/absence of diabetes mellitus, history of alcohol abuse, levels of albumin, alanine transaminase, aspartate aminotransferase, and total bilirubin, platelet count, prothrombin activity (PT), indocyanine green retention rate at 15 min (ICGR-15), and Child-Pugh score. Of tumor-related factors, the maximum tumor size, tumor number, tumor types, and sites of tumors were evaluated. Furthermore, serum concentrations of alpha-fetoprotein (AFP) and protein-induced by vitamin K absence or antagonist-II (PIVKA-II) were assessed. For the evaluation of tumor status, various criteria for liver transplantation in HCC, such as Kyoto criteria, 5–5-500 rule (Japan criteria), and up-to-seven criteria were used, because HCC beyond these criteria progresses to a systemic disease. The Kyoto criteria include the following standards: maximum tumor size ≤5 cm, tumor number ≤10, and PIVKA-II level ≤400 mAU/mL (Takada et al., 2007). The 5–5-500 rule sets criteria as follows: maximum tumor size ≤5 cm, tumor number ≤5, and AFP level ≤500 µg/L (Shimamura et al., 2019). In the up-to-seven criteria, the sum of the maximum tumor size (in cm) and the tumor number must be <7 (Mazzaferro et al., 2009). The outcomes and prognostic factors after HR were evaluated to develop new HR criteria.

*Statistical analysis*

The mean ± standard deviation of age and maximum tumor size and the median value and quartile range of other factors were evaluated. The survival rate was calculated by the Kaplan-Meier method using the log-rank test. Moreover, using the Cox proportional hazards model, multivariate analysis of factors with p<0.1 in the univariate analysis was performed to identify prognostic factors. The cut-off values of continuous variables, such as albumin level and tumor markers, were selected by receiver operating characteristic (ROC) curve analysis. In addition, we finally decided the cut-off values that were close to the most significant difference and easy to memorize and apply clinically (e.g., a cut-off value for albumin level of 35 g/L is also used in the Child-Pugh classification). P-values <0.05 were considered statistically significant. All statistical analyses were performed with R version 3.5.3 (The R Foundation for Statistical Computing, Vienna, Austria; https://cran.r-project.org/bin/macosx/).

## Results

The clinical characteristics of study patients are shown in Table 1. The average age was 70.1 ± 7.9 years, and 90 of 110 patients were men. Liver function was relatively good, with a median albumin level of 40 g/L and median PT activity of 91.6%; the median ICGR-15 value was 13.7%. However, only 14 (12.7%) patients with Child-Pugh B were included in the study. Moreover, the average maximum tumor size was 5.6 cm, and 66 patients had 2 tumors and 14 patients had more than 5 tumors. The median serum concentrations of AFP and PIVKA-II were 35.3 µg/L and 237 mAU/mL, respectively. Regarding operative factors, the median intraoperative blood loss and operation time were 665 mL and 307 min, respectively. Thirty-eight patients underwent blood transfusions and 29 patients had Clavien-Dindo grade ≥III complications (Dindo et al., 2004) (Table 1).

The results of univariate and multivariate analyses are shown in Table 2. Albumin level ≥35 g/L, AFP <100 µg/L, PIVKA-II level <100 mAU/mL, and maximum tumor size <5 cm were associated with a significantly improved prognosis in univariate analysis (p<0.05). Multivariate analysis was performed by using several factors of age <77 years old, albumin level ≥35 g/L, ICGR15 <20 %, Child-Pugh classification A, AFP <100 µg/L, PIVKA-II level <100 mAU/mL, and maximum tumor size <5 cm, which were p<0.1 in univariate analysis. The result showed that albumin level ≥35 g/L and AFP <100 µg/L were independent prognostic factors of HR in intermediate-stage HCC and that tumor size and tumor number were not independent prognostic factors in multivariate analysis (Table 2).

**Figure 1a, 1b, 1c:**
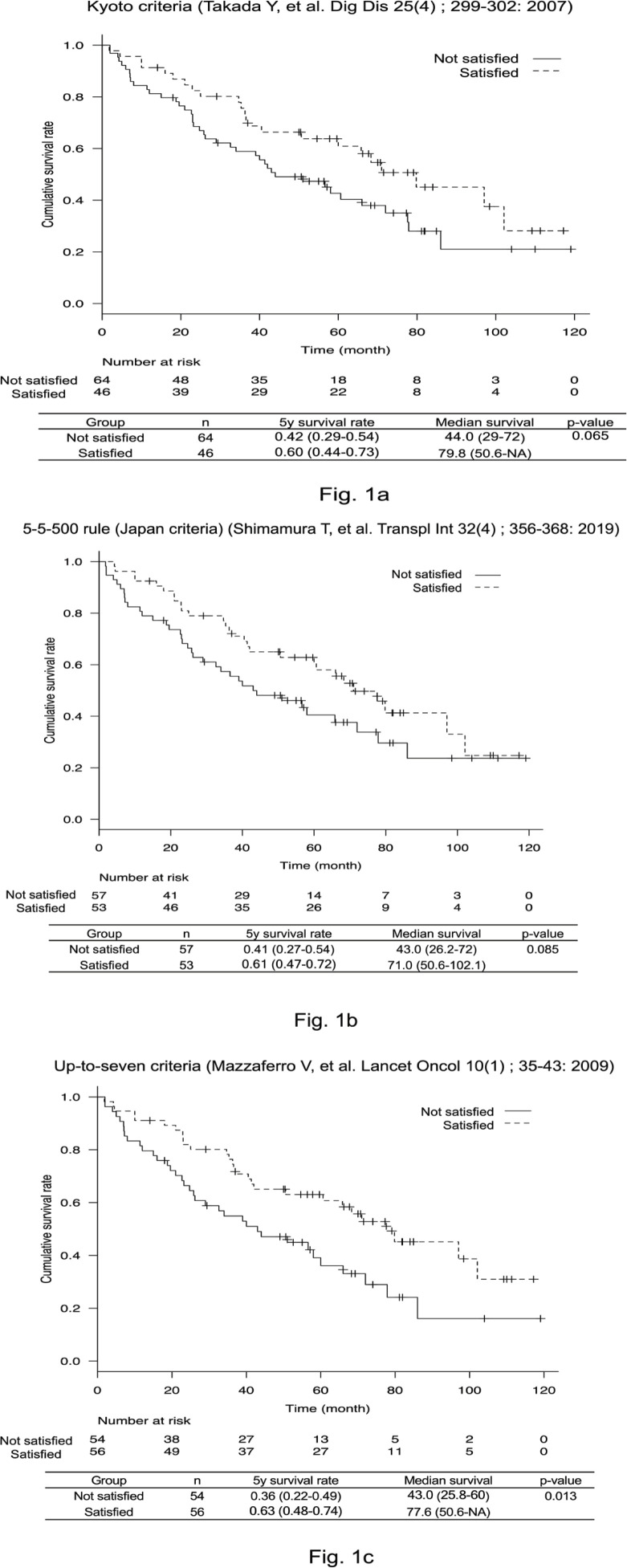
Survival Curves Using Different Liver Transplantation Criteria. The 5-year survival rates for the Kyoto criteria (Figure1a), 5-5-500 rule (Figure 1b), and up-to-seven criteria (Figure 1c) were 60%, 61%, and 63%, respectively. A significantly better prognosis was observed for the up-to-seven criteria (p = 0.013)

**Figure 2a, 2b, 2c:**
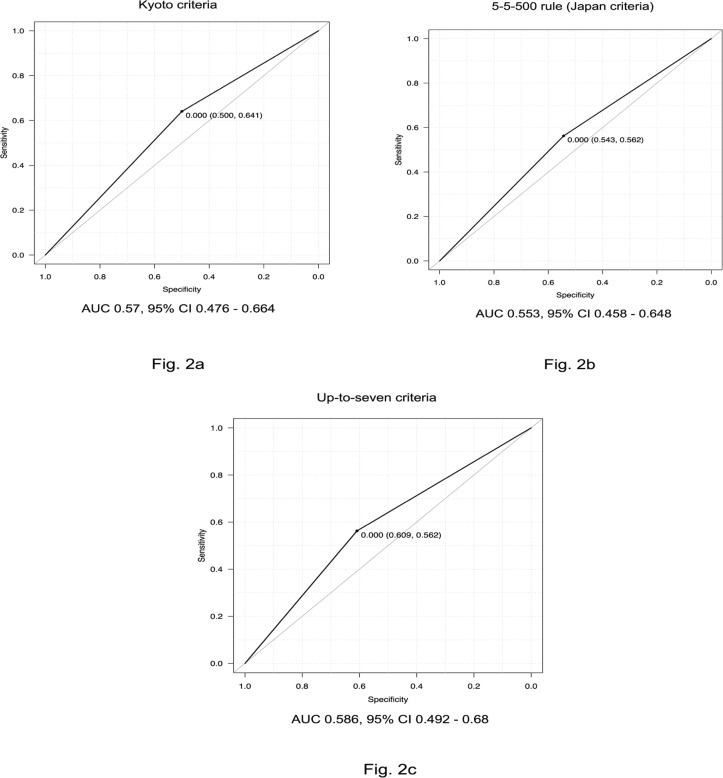
Receiver Operating Characteristic (ROC) Curves Using Different Liver Transplantation Criteria. The area under the curve (AUC) was calculated for each set of liver transplantation criteria (Kyoto criteria; Figure 2a, 5-5-500 rule; Figure 2b, and up-to-seven criteria; Figure 2c). The up-to-seven criteria had the best accuracy (AUC = 0.58)

Next, we performed examinations using various liver transplantation methods: the Kyoto criteria, 5-5-500 rule, and up-to-seven criteria. Patients who satisfied each of these criteria had good prognoses (Figure 1a, 1b, 1c). Forty-six of 110 patients satisfied the Kyoto criteria and had a 5-year survival rate of 60.9% (p = 0.065). For the 5-5-500 rule, 53 patients satisfied the criteria and showed a 5-year survival rate of 61.0% (p = 0.085). Although the number of patients who met the up-to-seven criteria was 56, the 5-year survival rate was 63.0% (p = 0.013). Further, the ROC curve and area under the curve (AUC) for each set of criteria were investigated; the up-to-seven criteria yielded the most favorable result (AUC = 0.586, Figure 2a, 2b, 2c).

**Figure 3a, 3b:**
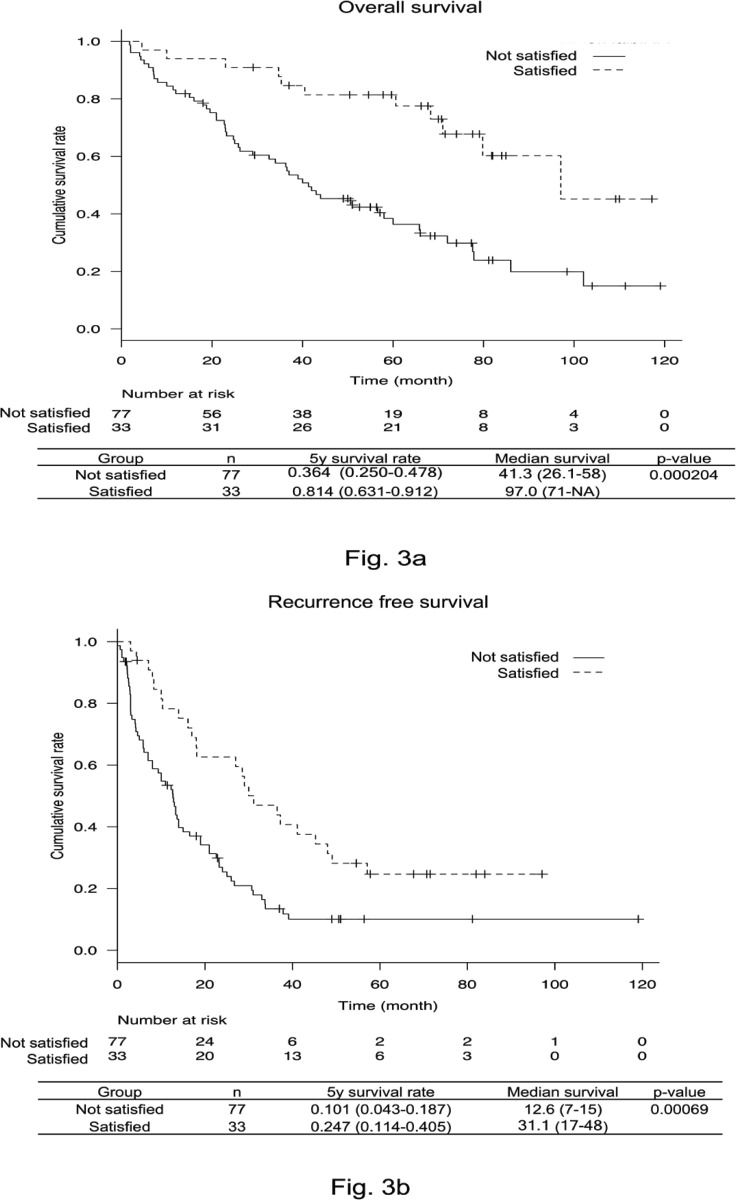
Overall Survival and Recurrence Free Survival Rate of the New Hepatic Resection Criteria. The 5-year survival rate for the new hepatic resection criteria (up-to-seven criteria, albumin level ≥35 g/L, and alpha-fetoprotein level <100 µg/L) was 81.4% (Figure 3a) and the 5-year recurrence-free survival rate was 24.7% (Figure 3b)

**Figure 4:**
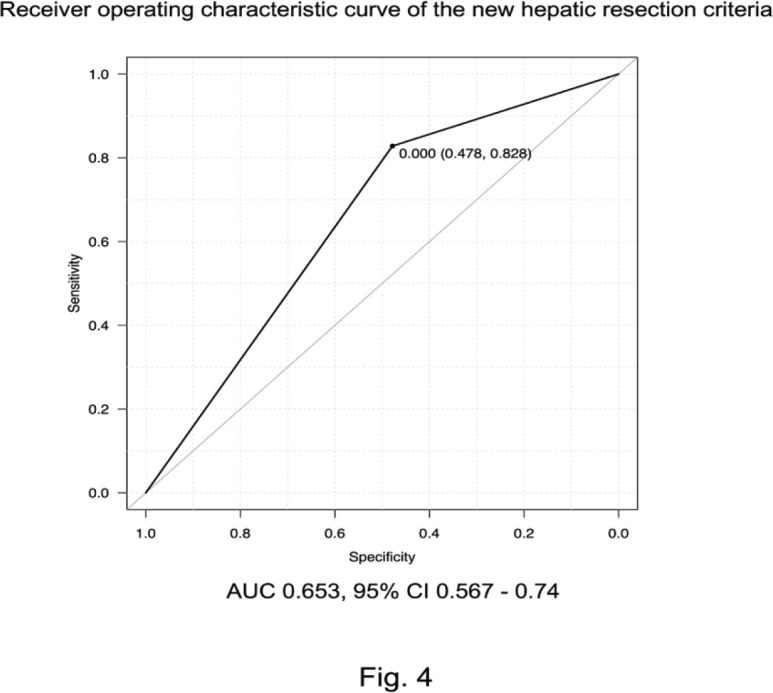
Receiver Operating Characteristic (ROC) Curves According to the New Hepatic Resection Criteria. The area under the curve (AUC) of the new criteria was 0.653, which was significantly better than for the Kyoto criteria, 5-5-500 rule, and up-to-seven criteria

New HR criteria were created using the above results and the prognosis was examined. The cumulative and recurrence-free survival rates were significantly higher in patients who satisfied the up-to-seven criteria, serum albumin level ≥35 g/L, and serum AFP level <100 µg/L (p = 0.000204, p = 0.00069, respectively). Moreover, the 5-year cumulative survival rate of patients who satisfied the new HR criteria was 81.4%. In addition, the 5-year recurrence-free survival rate was 24.7% (Fig. 3a, 3b). The AUC of patients satisfying these new HR criteria was 0.653, which was significantly better than for the Kyoto criteria, 5-5-500 rule, and up-to-seven criteria (vs. Kyoto criteria, p = 0.006; vs. 5-5-500 rule, p = 0.014; vs. up-to-seven criteria, p = 0.005; Figure 4).

**Table 1:** Clinical Characteristics of Study Patients

Factor		n=110
Age (year)		70.1 ± 7.9
Gender (%)	Female	20 (18.2)
	Male	90 (81.8)
Diabetes mellitus (%)		42 (38.2)
Etiology (%)	HBV	14 (12.7)
	HCV	63 (57.3)
	NBNC	33 (30.0)
Alcohol abuse (%)		44 (40.0)
Albumin (g/L)		40 [35, 42]
ALT (IU/L)		38 [25, 56]
AST (IU/L)		43 [29, 64]
Total bilirubin (µmol/L)		11.9 [8.5, 15.3]
Platelet count (x10^9^/L)		158 [121, 192]
Prothrombin activity (%)		91.6 [82.2, 101.2]
ICGR15 (%)		13.7 [9.4, 19.1]
Child-Pugh (%)	A	96 (87.3)
	B	14 (12.7)
Tumor size (cm)		5.61±3.75
Tumor number	2	66 (60.0)
	3	20 (18.2)
	4	10 (9.1)
	≥5	14 (12.7)
Tumor type (%)	Simple nodular	55 (50.0)
	Simple nodular + extra nodular	31 (28.2)
	Confluent multinodular	23 (20.9)
	Invasive	1 ( 0.9)
Tumor site (%)	Unilateral	75 (68.2)
	Bilateral	35 (31.8)
AFP (µg/L)		35.3 [7.9, 416.4]
PIVKA-II (mAU/mL)		237 [47, 5058]
Blood loss (ml)		665 [311, 1423]
Operation time (min)		307 [232, 376]
Operation method (%)	Anatomical	43 (39.1)
	Anatomical + partial	18 (16.4)
	Partial	49 (44.5)
Combination of ablation (%)	Resection only	96 (87.3)
	Resection + ablation	14 (12.7)
Intraoperative transfusion (%)		38 (34.6)
Complication (Clavien-Dindo, grade )	II	14 (12.7)
	IIIa	27 (24.6)
	IIIb	1 (0.9)
	IVa	1 (0.9)

ALT, alanine aminotransferase; AST, aspartate aminotransferase; ICGR15, indocyanine green retention rate at 15 minutes; AFP, α-fetoprotein; PIVKA-II, protein induced by vitamin K absence or antagonist-II; HBV, hepatitis B virus; HCV, hepatitis C virus; NBNC, negative of hepatitis B and hepatitis C virus; Age and tumor size are expressed as mean ± SD. Other data are expressed as median with 25th and 75^th^ percentiles.

**Table 2:** Univariate and Multivariate Analysis of Prognostic Factors

Factor	Group	n	Univariate analysis		Multivariate analysis	
			Median survival	p-value	Odds ratio (95% CI)	p-value
Age (year)	<77	87	68.3 (50.6-79.8)	0.0554	1.01 (0.98-1.05)	0.53
	≥77	23	34.0 (18-51)			
Gender	Female	20	79.8 (18-NA)	0.832		
	Male	90	58.0 (40-77.6)			
Diabetes mallitus	Negative	68	65.8 (36.5-79.8)	0.847		
	Positive	42	56.6 (37-86)			
HCV	Negative	47	68.3 (40-97.0)	0.79		
	Positive	63	58.0 (36.5-79.8)			
HBV	Negative	96	58 (40-72)	0.28		
	Positive	14	NA (8-NA)			
Alcohol abuse	Negative	66	43.0 (25.8-72)	0.166		
	Positive	44	68.3 (56.6-97.0)			
Albumin (g/L)	<35	21	25 (18.7-50.6)	<0.001	2.37 (1.15-4.89)	0.019
	≥35	89	71 (51-97.0)			
ALT (IU/L)	<30	43	43 (32.6-86)	0.604		
	≥30	67	66 (44-79.8)			
AST (IU/L)	<40	52	72 (43-97.0)	0.116		
	≥40	58	44 (24.7-68.3)			
Total bilirubin (µmol/L)	<12	47	77.8 (37-NA)	0.222		
	≥12	63	58.0 (36.5-71)			
Platelet count (x109/L)	<160	54	58.0 (37-86)	0.997		
	≥160	56	60.6 (36.3-79.8)			
Prothrombin activity (%)	<90	62	60.0 (37-79.8)	0.98		
	≥90	48	65.8(34-102.1)			
ICGR15 (%)	<20	83	71.0 (43-86)	0.0921	1.12 (0.63-2.01)	0.7
	≥20	27	40.5 (19.5-68.3)			
Child Pugh	A	96	66.0 (43-79.8)	0.0711	1.13 (0.46-2.73)	0.79
	B	14	33.5 (18-NA)			
Tumor type	Simple nodular	55	66.0 (41.3-79.8)	0.635		
	Others	55	50.6 (25-86)			
Tumor site	Unilateral	75	58 (36.3-77.6)	0.492		
	Bilateral	35	72 (37-97.0)			
AFP (g/L)	<100	62	86 (66-NA)	<0.001	2.83 (1.68-4.76)	<0.001
	≥100	48	34 (21-41.3)			
PIVKA-II (mAU/mL)	<100	39	79.8 (56.6-NA)	0.0373	1.40 (0.78-2.50)	0.26
	≥100	71	41.3 (32.6-71)			
Tumor size (cm)	<5	61	77.6 (50.6-102.1)	0.0326	1.33 (0.78-2.28)	0.29
	≥5	49	43 (25.8-65.8)			
Tumor number	<4	86	60.6 (41.3-79.8)	0.693		
	≥4	24	56.6 (23.2-NA)			

HBV, hepatitis B virus; HCV, hepatitis C virus; ALT, alanine aminotransferase; AST, aspartate aminotransferase; ICGR15, indocyanine green retention rate at 15 minutes; AFP, α-fetoprotein; PIVKA-II, protein induced by vitamin K absence or antagonist-II

## Discussion

In this study, we propose new HR criteria for patients with intermediate-stage HCC consisting of the up-to-seven criteria, serum albumin level ≥35 g/L, and serum AFP level of <100 µg/L.

Although TACE is the only recommended treatment for intermediate-stage HCC based on the BCLC guideline, several studies have demonstrated the necessity to modify the current treatment strategy (Kudo et al., 2015; Hiraoka et al., 2016; D’Avola et al., 2011; Ciria et al., 2015; Furukawa et al., 2017; Bell et al., 2017; Galle et al., 2017). Recently, several reports indicated the effectiveness of HR for intermediate-stage HCC. Observational data obtained from a study performed in 10 Western medical institutes on 2,046 consecutive patients with HCC who underwent HR suggested that HR would be effective for the treatment of patients with BCLC stage B or C HCC (Torzilli et al., 2013). Moreover, an investigation in Asia of 1,259 consecutive patients with BCLC stage B or C HCC who underwent HR (n = 908) or TACE (n = 351) demonstrated the effectiveness of HR (Zhong et al., 2013; Zhong et al., 2014). One randomized clinical trial (RCT) from China including patients with BCLC stage B and C HCC suggested that the prognosis of HR is significantly better than that of TACE (Yin et al., 2014). A meta-analysis of HR for intermediate-stage HCC including a total of nine studies with 4,958 patients also demonstrated a favorable hazard ratio of 0.433 (95% confidence interval [CI] 0.394–0.475) for 5-year overall survival compared to TACE (Liu et al., 2015). Similarly, in another meta-analysis, the overall survival rate (hazard ratio 0.60, 95% CI 0.55–0.66) was significantly higher in the HR group than in the TACE group (Qi et al., 2015). In a similar investigation of 18 high-quality studies, including one RCT, five propensity score-matching (PSM) studies, and 12 non-RCT and PSM studies, HR for BCLC stage B HCC demonstrated a significantly better survival rate (hazard ratio 0.53, 95% CI 0.43–0.65) (Hyun et al., 2018). Based on the aforementioned results, HR could improve the prognosis of some patients with BCLC stage B HCC. Several studies performed subgroup analyses to specifically determine which patients with intermediate-stage HCC could have an improved prognosis with HR. One study showed that HR could improve the prognosis of patients with Child-Pugh A HCC (Lin et al., 2010). Another report demonstrated more favorable survival rates in patients with fewer than three tumors who underwent HR; however, no significant difference was found between HR and TACE for patients with more than three tumors (Jianyong et al., 2014). 

Recently, the need for HR criteria for intermediate-stage HCC has been advocated. Tada et al. concluded that HR in patients with intermediate-stage HCC contributes more significantly to prognosis improvement than TACE in patients with a Child-Pugh score of 5 and three or fewer tumors (Tada et al., 2017). Wada et al., (2016) subdivided patients with intermediate-stage HCC according to tumor number and size. Patients were classified into three groups based on radiological findings regarding tumor number and maximum tumor diameter: type 1, up to 3 lesions <5 cm; type 2, up to 3 lesions ≥5 cm or 4 nodules of any size; and type 3, >4 nodules. They concluded that type I is an appropriate indication of HR. Kamiyama et al., (2017) demonstrated that BCLC stage B HCC should be divided based on the serum concentration of AFP multiplied by that of PIVKA-II to determine the patients that would benefit from HR. Thus, the criteria proposed by Tada et al., (2017) included tumor number and liver function, those proposed by Wada et al., (2016) included tumor number, tumor size, and tumor location, and those proposed by Kamiyama et al., (2017) included tumor number and tumor markers as malignant potential. The criteria in this study included the up-to-seven criteria, which include tumor number and size, albumin concentration as a measure of liver function, and tumor marker (AFP) as malignant potential. Therefore, the new criteria in this study cover all factors used as markers in previous reports. The 5-year cumulative survival rate in previous studies was 54.2% (Tada et al., 2017), 75.2% (Wada et al., 2016), and 69.5% (Kamiyama et al., 2017). The 5-year cumulative survival rate based on the new criteria in this study was 81.4%, which indicates that the new criteria can select more favorable candidates for HR from patients with the intermediate-stage HCC. Furthermore, the 5-year cumulative survival rate in this study was better than that of HR for multiple HCC within ≤3 cm and ≤3 nodules reported by a recent Japanese nationwide survey (70.7%) (Fukami et al., 2019).

In this study, we used various liver transplantation criteria to determine the criteria for resection of intermediate-stage HCC. These criteria consisted of different combinations of tumor size, number, and malignant potential. When these criteria are exceeded, the disease is considered to have become systemic. Similarly, Kudo et al., (2015) and Yamakado et al., (2014) evaluated the criteria for molecular targeted drugs and TACE not by tumor number or size alone, but in combination. Therefore, the up-to-seven criteria (a combination of these factors), are an important indicator for intermediate-stage HCC. In addition, tumor number and tumor size were examined in multivariate analyses. As a result, only albumin level ≥35 g/L and AFP level <100 µg/L were significantly prognostic. Since the up-to-seven criteria contain these overlapping factors (tumor number and size), they were not included in multivariate analysis.

The main points of this paper are that the up-to-seven criteria (the combination of tumor size and number), AFP level as malignant potential, and albumin level as liver function can be used to select patients with intermediate HCC for HR. 

The AUC when the three factors of up-to-seven criteria, albumin level ≥35 g/L, and AFP level <100 µg/L were satisfied was 0.653 (95% CI 0.56–70.74), which was significantly better than the three AUCs shown in Figure 2. Of course, there may be various treatments after recurrence, but it is useful that the criteria in this study can predict the prognosis and determine the indication for HR regardless of those treatments.

The novelty of the HR criteria for intermediate-stage HCC revealed in this study was to utilize the criteria for liver transplantation. The new criteria might be practical and meaningful because HCC becomes a systemic disease if it exceeds the criteria for liver transplantation.

This study has some limitations. The subjects in this study were selected from patients with intermediate-stage HCC patients because HR was the only treatment option. In addition, the results of HR were not compared with those of other treatments such as TACE. This study was a retrospective study with a relatively small number of patients. Therefore, further studies with larger numbers of patients, including patients who underwent other treatment modalities, are necessary. The need for validation of criteria due to advances in chemotherapy is an important limitation of this study. In addition, as described above, only one RCT for intermediate-stage HCC between HR and TACE has been performed to date. Therefore, since some background selection biases in meta-analysis cannot be avoided, a high quality RCT is needed in the future.

In conclusion, HR can improve the prognosis of patients with intermediate-stage HCC who satisfy the new HR criteria consisting of the up-to-seven criteria, serum albumin level >35 g/L, and serum AFP level <100 µg/L. 
